# The Effect of Lockdown Due to the COVID-19 Pandemic on Digital Eye Strain Symptoms Among the General Population: A Cross-Sectional Survey

**DOI:** 10.3389/fpubh.2022.895517

**Published:** 2022-06-22

**Authors:** Mohammad Abusamak, Hatim M. Jaber, Hamzeh Mohammad Alrawashdeh

**Affiliations:** ^1^Department of Surgery, School of Medicine, Al-Balqa Applied University, Amman, Jordan; ^2^Department of Public Health, Amman Eye Clinic, Amman, Jordan; ^3^Department of Community Medicine, School of Medicine, Al-Balqa Applied University, Amman, Jordan; ^4^Ophthalmology Department, Sharif Eye Centers, Amman, Jordan

**Keywords:** symptoms, digital eye strain, general population, COVID-19, neck pain

## Abstract

Repetitive prolonged use of digital devices without regular breaks has detrimental effects on ocular health and quality of vision. Individuals with chronic eye problems and refractive errors are at higher risk of developing digital eye strain (DES). Correction of refractive errors, adopting healthy practices will reduce its risk. The survey examined the effect of prolonged lockdown on the development and increased severity of digital eye strain (DES) symptoms among the general population. An online survey was conducted in March 2020 on social media platforms in Jordan. Data from 1,460 responders were analyzed. About half of the respondents were between 30–49 years, 28.4% were retired, 21% had chronic systemic illness, and 23% reported chronic eye problems. A rise in the use of digital devices during lockdown was reported by 957 participants, with 33% of them used digital devices more than 4 h a day. The most common symptoms before and during lockdown were headache and neck / shoulder pain have the highest associations (*X*^2^ = 280.0, 271.3, df = 4, *p* < 0.001 respectively). Female gender, existence of chronic eye problems and systemic diseases, and duration of using digital devices were found to be statistically significant factors associated with increasing severity of eye symptoms during lockdown. Not taking enough regular breaks from digital devices showed significant association with blurred vision at distance and near (*X*^2^ = 13.03, 10.74, *df* = 4, *P* = 0.011, 0.03, respectively). People with chronic eye problems and increased time using devices during lockdown developed new eye complaints three times more than before the lockdown and males were two times more likely than females to have more severe eye symptoms.

## Introduction

Digital eye strain (DES), frequently referred to as computer vision syndrome (CVS) or visual tiredness, is a cluster of ocular, vision-related, and musculoskeletal symptoms caused by prolonged use of digital devices (also known as video display terminals [VDTs]). A “digital device” is any electronic hardware that is used on a daily basis and may include a variety of devices such as cell phones, smart wristwatches, desktop computers, tablets, virtual reality viewers, 3D displays, and e-readers. The most common symptoms of DES are eye pain, dry eyes, headaches, blurred vision, and neck and shoulder pain (1, 2). Due to the global shift in the usage of digital devices over the last few decades, DES has emerged into a very real and identifiable problem affecting millions of individuals and exposing individuals of all ages at risk (1, 2).

The repeated use of digital devices for more than two continuous hours is putting the user at a great risk of developing DES due to the excessive accommodative demands (1, 3). Refractive errors that are uncorrected, under-corrected, or over corrected can exaggerate the symptoms (4). Unlike printed pages, letters on digital displays are not sharply outlined, with weaker letters' contrast to the background, as well as the effect of glare and reflection, rendering viewing more difficult (5). Furthermore, given the various distances and angles of viewing, the eye movement and focusing demands are substantially higher than those needed when writing on or reading from paper. Muscle spasms and pain can occur as a result of poor posture when using digital screens, especially in the neck, shoulders, and back. This is particularly evident in people with refractive errors and substandard viewing glasses or contact lenses, which cause them to tilt their heads or lean on the screen in awkward ways in order to see more clearly (5). The majority of people develop DES when the visual demands of performing tasks overcome their visual capability to do so comfortably (6).

The DES conveys external symptoms similar to dry eye disease (DED), including ocular pain, foreign body sensation, tearing, burning, and heaviness of eyelids. Among other symptoms, extended use of digital devices exacerbates these symptoms. It is critical to distinguish DES from DED, despite the fact that they share similar symptoms and strongly associated (7). The DES presents symptoms that are both internal (e.g., blurred vision, headache, diplopia) and external in nature (e.g., neck and shoulder pain, foreign body sensation, burning sensation, photophobia) and are directly related to the excessive and repetitive use of VDTs (8). Adults are more prone to develop DED, which is caused by a variety of factors, as demonstrated by tear film instability induced by ocular conditions (9, 10).

Digital eye strain management is not simple. It requires the treatment of prior eye problems such as ocular surface disease and the optimal treatment of current eye symptoms by taking frequent breaks and adjusting the way video digital screens are presented by following healthy positions of posture (11–14). In addition, the correction of refractive errors, especially spherical hyperopia and astigmatism, is associated with better outcomes (4).

The spread of COVID-19 was rather troubling to health authorities and the public at large in early February 2020. Several countries agreed to suspend foreign and domestic travel and enforce curfews on their citizens for several weeks to slow the spread of the pandemic. As a result of this situation, people were forced to rely on the internet and digital devices as their primary means of communication. The authors found that prolonged use of digital devices, particularly by students and academics, appeared to be associated with an increase in eye symptoms, which inspired the concept for this survey. As a result, this survey aims to examine how lockdown has affected DES symptoms in the general population. An association was expected between occupation, age, gender, and the prevalence of chronic systemic and ocular disease, which would affect the emergence of new symptoms and increase the severity of DES symptoms (1, 3). We believe that the novelty of our study was in its objectives as it assessed the emergence of new symptoms due to the restrictive measures imposed on the general population.

## Methods

Between March 26 and April 29, 2020, an online, anonymous, cross-sectional, observational study was conducted. It consists of a self-administered web-based questionnaire conducted using social media platforms. Inclusion criteria were residency in Jordan, being 18 years of age or older, and a consent to participate. As a consequence, questions on age and desire to participate were included to the questionnaire at the beginning. The data collection started 7 days after the Jordanian lockdown began (March 19, 2020) and ended a few days before the lockdown ended to guarantee that digital eye strain symptoms are distinct from other stress-related symptoms such as headaches.

### Ethical Considerations

Ethical approval was obtained from the Institutional Review Board of the Al-Balqa Applied University (BAU) abiding by the tenets of the declaration of Helsinki 1975 and its amendments in 2008 and later. The purpose of the study was clearly explained in the opening page of the survey and voluntary participation was encouraged. No personal information was obtained, and the confidentiality of the data was assured.

At the first part of the online survey, all participants were required to sign an electronic informed consent form that included extensive information on the study goals, objectives, methods, supervisor contact information, and IRB approval. Additionally, participants were instructed that participation was voluntary and that they might stop the survey at any stage. The data were kept confidential because all identifiable information was stripped and no identifier-related questions such as participant name, city of residence, or zip code were requested. Additionally, a study-specific unique identifier was generated for each participant, and this file was locked and password-protected with controlled access and authorizations for viewing, sharing, and using it reserved to the research team. All further analyses were conducted on this anonamyzed file. Participants did not receive any compensation or rewards for their participation in the study.

### Jordan Population and Online Sampling Process

Non-probability sampling methods were used for the online survey, such as convenience sampling, volunteer opt-in panels, and snowball sampling. In general, a sample size of around 10% of the population, but not exceeding 1,000 participants, was considered appropriate. The total population of Jordan was estimated to be 10.5 million in 2020, with about 1.3 million Syrian refugees residing in the country. The authors computed a sample size using a prevalence of digital eye strain of 50% as the most judicious estimate with a margin of error of 2.5 % [95 percent confidence interval (CI): 47.5–52.5 percent]. Initially, the sample size (*n*) of 1,537 was calculated using the following formula: n = N x/[(N-1) E2+x]. Where N is the population size (10,500,000), E is the margin of error (2.5%), r is the frequency of digital eye strain (50%), and Z is the critical value for the confidence level (5%). After cleaning the dataset from incoherent and incomplete responses, the final sample size used was 1,460 (15).

To achieve a high response rate, demographic segmentation and social networks had been utilized to gather data from the respondents in universities, colleges, and social groups, as well as educators and university professors. To reach the target demographic, the survey link was posted on Facebook and other social media platforms favored by the participants, and they redistributed the survey via email, Messenger, and WhatsApp.

### The Survey Instruments

The survey was online self-administered using Microsoft forms. The questionnaire instrument was developed based on literature review and frameworks similar studies (8, 16–19). It was originally written in English and then translated into Arabic, Jordan's official language. Afterwards, it was re-translated into English by two independent translators and compared by a third. Validity was tested in a pilot study with 30 randomly self-selected participants. The pilot study confirmed that the survey results were significant and could be administrated online.

The first introductory section emphasized the study's purpose and objectives, as well as a consent form. It was composed of three parts of closed-ended single- and multiple-choice questions; symptoms were graded using an adjectival scale. Part one of the survey covered screening questions to ensure that the expected target respondents were reached. This includes questions about age, cis-gender, and activity, as well as the existence of chronic systemic diseases such as diabetes mellitus, systemic hypertension, hyperlipidaemia, neurological disorders, and allergies. The second part examined how many hours per day were spent in front of screens, including television, phones, and computers, in the 2 weeks preceding the lockdown's start. The amount of hours spent watching digital gadgets was a multiple-choice question that ranged from 2 h to more than 10 h per day. The second part of the survey included additional exploratory questions about the presence of eye disorders before to the lockdown, including dry eye disease, allergic eye disease, cataract, glaucoma, age-related visual impairments, and retinal disorders. Finally, a three-point scale question was asked about the habit of taking regular breaks while using digital devices.

**Figure 1:**
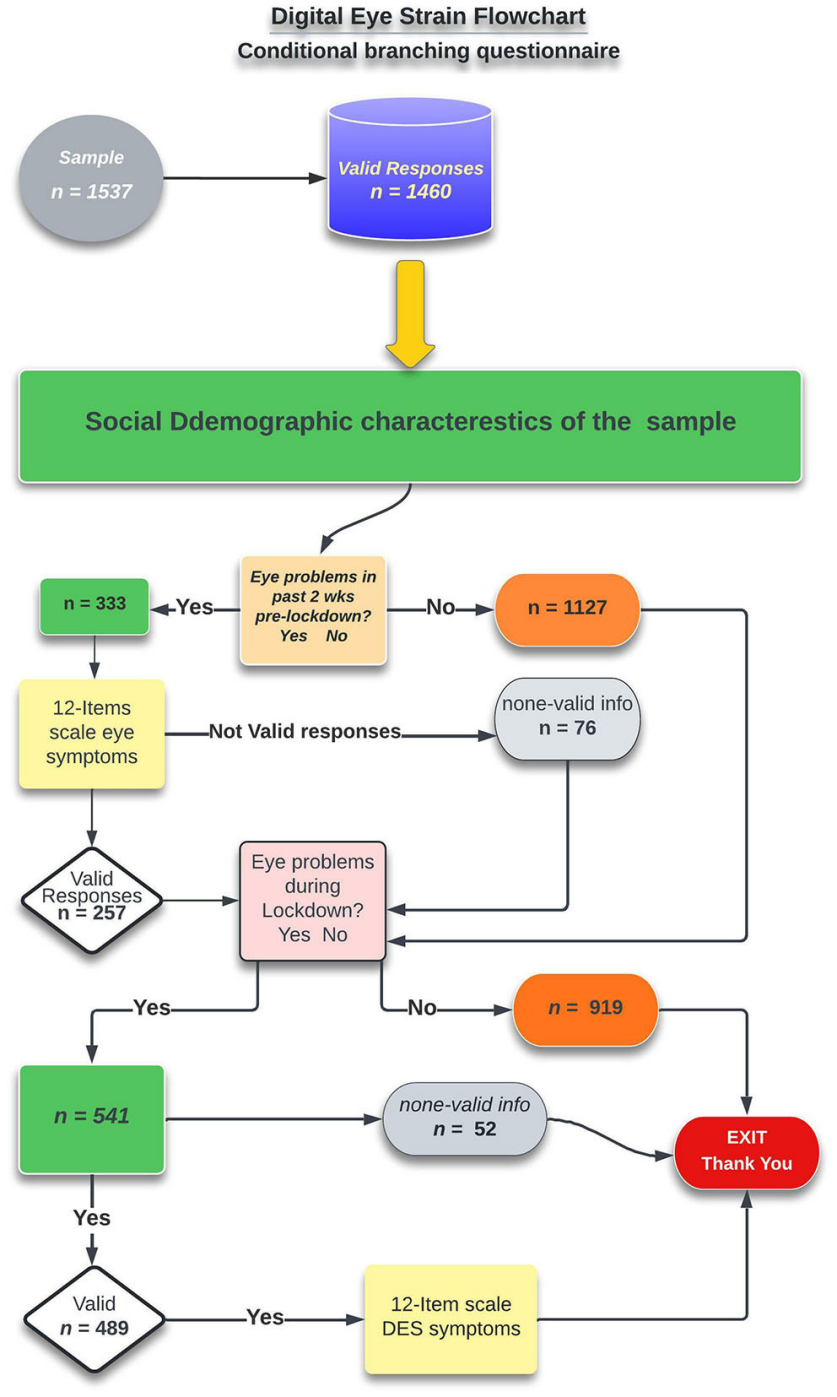
Flowchart depicting the organization of the survey questions and their conditional branching.

The final section of the survey investigated the influence of lockdown on the specific symptoms associated with digital eye strain. Following a brief introduction, a yes/no questions were presented to determine whether the subject experienced an emergence and increase in ocular symptoms during the lockdown. If the subject denied any development or increase in the symptoms severity, the survey was terminated. If the respondent indicates yes, he or she will be asked to rate their symptoms on a scale of absent to always present. The following diagram illustrated the survey conditional branching processes and participants numbers in each arm of the study (Figure 1).

A 12-item scale was used twice to assess the DES: the first time to determine the presence of those symptoms at the baseline (i.e., the weeks before the lockdown started) and the second time to determine the severity and the emergence of new symptoms. These included blurred vision at both distant and near distances, a burning sensation, redness, lacrimation, heaviness of the eyelids, eye discomfort (foreign body sensation), double vision, eye pain, headaches, photophobia, and neck / shoulder pain. To enable easier use by the public, the authors used a three-point adjectival scale (not present, occasionally present, and always present) rather than the standard five-point Likert scale. Finally, the authors examined how much time people spend on digital devices in two ways: how much time they spend on digital devices on a daily basis and and the amount of extra time they spend each day.

### Data Management and Analysis

The data were collected using Microsoft® Forms and downloaded into Excel® for coding and de-identification before being imported into the Statistical Package for Social Science (IBM SPSS Statistics for Windows, version 25, IBM Corp., Armonk, N.Y., USA) for analysis.

### Descriptive Statistics

A descriptive analysis was conducted to identify the statistical validity of all variables, and frequencies and percentages for the sample demographic characteristics and ocular and muscular symptoms were calculated. Cronbach's alpha (α) was used to determine the internal consistency reliability of the adjectival scales items. Several Cronbach's assumptions were examined and found to be consistent for each scale developed, including that the items on the scales were ordinal and the scales were unidimensional. The dependent variables in this study were the emergence of new eye complaints and the exacerbation of existing DES symptoms. A bivariate analysis was performed to evaluate the link between categorical variables (age groups, activity, and duration using digital devices). The logistic top-down step regression method was used to identify the relationship between sociodemographic health parameters and the occurrence of the two dependent variables; Odds Ratios (OR), also termed exp (B) in logistic regression output, 95% confidence intervals (95% CI) were reported where appropriate to indicate risk of developing ocular and/ or muscular symptoms. To investigate the existence of statistical correlations between demographic characteristics and ocular and muscular symptoms, a Chi-square test was used. For categorical variables, the chi-square test was used. Statistical significance was defined as a *p*-value of < 0.05.

## Results

Table 1 summarizes the sample's statistical distribution. The total sample size was 1,460 individuals, with a 5% rejection rate due to incomplete responses. Males comprised 59.5 % of interviewees. The majority the sample was between the ages of 30 and 49. Additionally, we found that the mean male age was 36.5 [±] 1.2 years, while the average female age was 39.4 [±] 1.5 years.

**Table 1:** Descriptive statistics of the sample.

**Variable**	**In % of total sample** **(*n* = 1,460)**
**Gender**	
Male	59.5
Female	40.5
**Age groups (years)**	
17–5	2.7
18–29	29.2
30–49	47.9
50–99	20.2
**Activity**	
School student	3
University student	16.6
Technician	7.5
Health worker	13
Clerical service worker	19.7
Teachers	11.9
Retired	28.4
**Chronic disease**	
Yes	20.9
No	79.1
**Chronic eye disease**	
Yes	22.9
No	77.1
**Baseline time using digital devices (hours/day)**	
(1–2)	19.3
(3–5)	42.2
(6–8)	19.9
> 8	18.6
**Increased time during lockdown (hours/day)**	
= 0	35.5
<1	4.7
(1–2)	21.1
(3–4)	18
>4	21.8

The majority of survey respondents were retired, constituting 28.4 percent of the total sample. According to the same table, 20.9 % of respondents had chronic diseases, while 22.9 % had chronic eye diseases. Prior to the lockdown, respondents reported spending an average of 5 [±] 2.6 h each day using digital devices. However, 957 respondents (65% of the total sample) reported that the lockdown increased their daily time spent on digital devices. The average daily increase in time was estimated to be 4 h (33 %).

### Quality and Reliability of the Scale Used

The internal consistency of the adjectival scale for DES symptoms was tested using factor analysis, and the 12 items had a Cronbach's alpha of 0.812. Kaiser-Meyer-Olkin (KMO) sampling adequacy was 0.848 (It is recommended to pass the 0.7 level). Additionally, Bartlett's sphericity test result was 712.5 with a degree of freedom of 66 and < 0.001 indicating significance.

### Prevalence and Severity of DES Symptoms

The prevalence and severity of the 12 DES symptoms were evaluated using the survey instrument prior and during lockdown. The most common symptoms before lockdown were neck and shoulder pain, headaches, blurred vision at a distance, burning sensation, blurred vision of near objects, and photophobia. The most common symptoms reported during the lockdown were neck and shoulder pain, headaches, burning sensation, blurred vision at a distance, photophobia, blurred vision of near objects, lacrimation, and eye strain as seen in Table 2.

**Table 2:** Self-reported symptoms prior and during lockdown (value in % of total sample) with Chi-square test of independence.

**Symptoms**	**Prior lockdown**		**During lockdown**			
	**Mild/Moderate**	**Severe**	**Mild/Moderate**	**Severe**	**X^**2a**^**	**P-value**
	***n* (%)**	***n* (%)**	***n* (%)**	***n* (%)**		
**I. Accommodative symptoms**						
Neck pain/Shoulder Pain	135 (9.2)	66 (4.5)	224 (15.3)	166 (11.4)	271.3	<0.001
Blurred vision at a distance	171 (11.7)	20 (1.4)	277 (19.0)	61 (4.2)	144.5	<0.001
Headache	146 (10.0)	44 (3.0)	249 (17.1)	109 (7.5)	280.0	<0.001
Blurred vision of near objects	131 (9.0)	55 (3.8)	193 (13.2)	123 (8.4)	202.4	<0.001
Eye strain	131 (9.0)	25 (1.7)	228 (15.6)	39 (2.7)	178.6	<0.001
Double vision	67 (4.6)	14 (1.0)	110 (7.5)	29 (2.0)	223.6	<0.001
**II. Dry eye symptoms**						
Burning sensation	163 (11.2)	24 (1.6)	302 (20.7)	55 (3.8)	151.1	<0.001
Photophobia	142 (9.7)	40 (2.7)	230 (15.8)	98 (6.7)	234.9	<0.001
Heaviness of eyelids	137 (9.4)	28 (1.9)	123 (8.4)	69 (4.7)	139.4	<0.001
Lacrimation	134 (9.2)	21 (1.4)	233 (16.0)	48 (3.3)	180.4	<0.001
FB sensation	106 (7.3)	15 (1.0)	181 (12.4)	36 (2.5)	225.8	<0.001
Eye redness	111 (7.6)	4 (0.3)	178 (12.2)	33 (2.3)	191.2	<0.001

^a^*, Chi Square*.

The results of the logistic regression to study the factors associated with new eye complaints (model 1) and increased severity of current eye complaints (model 2) are reported in Table 4. Both models have a satisfactory goodness-of-fit according to the Chi-square test (*p* < 0.0001) and respectively *R*^2^ = 9 and 14%.

The variables of chronic eye problems and increased time using digital devices during lockdown were found to be associated with the emergence of new eye complaints in model 1 in Table 4. The variable of chronic eye problems was significantly associated with having new eye complaints (*p* < 0.002). In comparing individuals with chronic eye problems to those who have not had chronic eye problems, the odds ratio shows that having new eye complaints after lockdown increased by around three times. The variable of increased time spent using digital devices during lockdown is significantly associated with the development of new eye complaints (*p* < 0.001). Furthermore, the odds ratio of 3.56 indicates that anyone who spent more time on digital devices had four times the risk of developing a new complaint as the person who did not spend time on them. In model 2, it was found that males developed severe eye symptoms two times more than females (*p* < 0.001). Odds ratios for the variables of chronic eye disease, increased time using digital devices during lockdown, and increased amount of time are higher than the odds ratios before lockdown, similar to model 1.

## Discussion

The government of Jordan imposed a strict lockdown and curfew hours for 6 weeks, beginning March 18, 2020 and ending April 29, 2020 to contain the COVID-19 pandemic. This study aimed to see how homebound lockdown affected the development and severity of baseline and new digital eye strain symptoms (DES).

The data revealed that young males responded to the online survey more than females, which was consistent with consumer behavior on social media platforms during the lockdown (20). This could have increased the prevalence of eye symptoms in male subjects, particularly the new onset of DES symptoms.

Females had more severe lockdown symptoms than males in this study, which were statistically significant for most symptoms such as neck/shoulder pain, photophobia, blurred vision at distance, eye redness, heavy eyelids, and difficulty focusing on near objects, as shown in Table 3.

**Table 3:** Sample characteristic factors associations with emergence of new DES symptoms and increased severity of existed symptoms.

	**Association of emergence of new eye symptom**				**Association of increased eye symptom severity**			
**Factor analyzed**	**No**	**Yes**	**X^**2**^**	**P-value**	**No**	**Yes**	**X^**2**^**	**P-value**
	***n* (%)**	***n* (%)**			***n* (%)**	***n* (%)**		
**Gender**					
Female	186 (12.7)	405 (27.7)	10.269	<0.001*	102 (7.0)	190 (13.0)	5.374	0.02*
Male	341 (23.4)	528 (36.2)			151 (10.3)	84 (5.8)		
**Age (in years)**				
<18	7 (0.5)	32 (2.2)	3.059	0.383	4 (0.3)	3 (0.2)	1.460	0.692
18–29	170 (12.0)	257 (17.6)			85 (5.8)	85 (5.8)		
30–49	247 (16.9)	452 (31.0)			112 (7.7)	135 (9.2)		
50 +	103 (7.1)	192 (13.2)			52 (3.6)	51 (3.5)		
**Activity**								
School student	9 (0.6)	35 (2.4)	16.908	0.01*	6 (4.1)	3 (0.2)	5.098	0.531
Univ. student	105 (7.2)	137 (9.4)			54 (3.7)	51 (3.5)		
Technician	46 (3.1)	63 (4.3)			25 (1.7)	21 (1.4)		
Health worker	60 (4.1)	130 (8.9)			26 (1.8)	34 (2.3)		
Clerical worker	83 (5.7)	204 (14.0)			43 (2.9)	40 (2.7)		
Teachers	63 (4.3)	63 (4.3)			26 (1.8)	37 (2.5)		
Retired	161 (11.0)	161 (11.0)			73 (5.0)	88 (6.0)		
**Chronic systemic disease**			
Yes	117 (8.0)	410 (28.1)	0.451	0.502	50 (3.4)	207 (14.1)	1.675	0.196
No	188 (12.9)	339 (23.2)			203 (13.9)	67 (4.6)		
**Chronic eye disease**								
Yes	182 (12.5)	158 (10.8)	18.42	<0.001*	57 (4.0)	100 (6.8)	11.481	0.001*
No	741 (50.8)	376 (25.8)			196 (13.4)	174 (11.9)		
**Time using digital devices before lockdown in hours/day**			
1–2	190 (13.9)	95 (6.5)	4.824	0.185	47 (3.2)	47 (3.2)	5.354	0.148
3–5	399 (27.3)	218 (14.9)			93 (6.4)	123 (8.4)		
6–8	174 (11.9)	110 (7.5)			51 (3.5)	54 (3.7)		
>8	161 (11.0)	113 (7.7)						
**Taking regularbreaks**								
No	22 (1.5)	24 (1.6)	2.965	0.227	9 (0.6)	15 (1.0)	0.025	0.988
Sometimes	95 (6.5)	91 (6.2)			33 (2.3)	58 (4.0)		
Always	61 (4.2)	40 (2.7)			15 (1.0)	25 (1.7)		
**Time increase using digital devices**								
Yes	710 (48.6)	247 (16.9)	21.555	<0.001*	253 (17.3)	27 (1.8)	429.26	0.001*
No	280 (19.2)	223 (15.3)			0.0 (0.0)	247 (16.9)		
**Time using digital devices during lockdown (hours/day)**								
= 0	503 (34.5)				503 (34.5)			
<1	58 (4.0)	10 (0.7)	31.091	<0.001*	58 (4.0)	10 (0.7)	30.833	0.001*
1–2	254 (17.4)	53 (3.6)			254 (17.4)	53 (3.6)		
3–4	191 (13.0)	72 (5.0)			191 (13.1)	72 (4.9)		
> 4	207 (14.2)	112 (7.7)			207 (14.2)	112 (7.7)		

*Indicates statistically significant values.

Correlation between gender and musculoskeletal symptoms was explored. We found that females had more neck/shoulder pain during lockdown than prior to lockdown (Table 2). For instance, in addition to the time spent on social media; women are helping their children in online school learning. An effective policy is needed to rationalize and to impose regular breaks women use of social media and online learning platforms (21).

The majority of the survey respondents declare using digital devices more than an average of 5 h daily. In addition, this finding is consistent with the 65% (*n* = 957) persons that have reported an increase in their use of digital devices from which 21.8% of them reported 4 h or more during the lockdown period as depicted in Table 1. As people self-reported their time spent on digital devices, it could be expected that they underestimated their actual time spent on digital devices because they may not have counted internet, TV, mobile phones, or tablets. Recent studies have found a global trend toward spending more time with digital devices, particularly among younger generations (16, 17, 22). In 2016, Common Sense Media (CSM) reported that American parents of teenagers spend about 9 h per day on the internet (23). Similarly, a survey conducted by CMS in 2019 revealed that teenagers spend more than 7 h per day on media (24). According to Reddy et al. (18), using digital devices for more than 2 h per day has a significant impact on DES symptoms (18). Blatter et al. (19) also observed that increased computer use, with or without mouse use, was correlated with musculoskeletal pain and dysfunction. Moreover, they found positive associations with work-related upper limb disorders for both genders with computer use of more than 6 and 4 h per day, respectively (19).

In the current study, people who do not taking regular breaks were found to have strong association with blurred vision at a distance and difficulty focusing on near objects. However, this is not so for neck pain or dry eye symptoms, respectively. Logaraj et al. (25) showed that students who took regular breaks were less likely to show symptoms of DES (25). Indeed, Lemma et al. (26) studied the effect of taking regular breaks on the development of DES when compared to those who did not take frequent breaks. It was found that secretaries who took regular breaks were 72.1 % less likely to experience digital eye strain (26).

Jordanian schools and universities rushed to adopt online education during the lockdown, resulting in a significant shift in the digital device usage habits of educators and students alike. Due to the compulsory online studies and high demand for internet, teachers, researchers and workers were among the most affected by new DES complaints during the lockdown (Table 3). This is in line with the findings of several studies conducted in Middle Eastern and Asian countries (17, 18, 22). Contrary to the hypothesis, technicians developed new symptoms of DES more than other activities (OR 1.42, 95 % C.I). The reason for this is that manual workers used digital devices for communication and online services more than they did previously. Interestingly, the lockdown resulted in an increase in the severity of symptoms reported by all respondents in the activity groups, with retired people having the highest odds ratio (OR 6.09, 95 % C.I.) followed by university students (OR 5.6, 95 % C.I.), which highlights the importance of public awareness and early management of DES in improving the education process and helping retired people improve their vision quality as seen in Table 4. Furthermore, retired people are at greater risk for DES because they are more likely to have chronic systemic and ocular diseases that were worsened by having to use digital devices for increased hours than they were when they first started (21).

**Table 4:** Factors associated with emergence of New Eye complaints and increased severity of current eye complaints by logistic regression.

**Factor analyzed**	**Model 1: New complaint development**		**Model 2: Increased severity**	
	**OR (95% CI)**	***p*–value**	**OR (95% CI)**	***p*–value**
**Gender**				
Female	1		1	
Male	1.30 (0.99–1.69)	0.051	1.78 (1.28–2.50)	0.001*
**Age (in years)**				
<18	1		1	
18–29	3.23 (0.48–21.60)	0.226	0.54 (0.03–9.43)	0.676
30–49	3.33 (0.49–22.70)	0.219	0.58 (0.33–10.08)	0.706
50 +	2.99 (0.22–0.67)	0.269	0.43 (0.02–7.58)	0.561
**Activity**				
School student	1		1	
University student	1.47 (0.25–8.06)	0.695	4.73 (0.28–79.88)	0.281
Technician	1.42 (0.24–8.45)	0.579	5.60 (0.31–97.52)	0.238
Health worker	0.93 (0.16–5.34)	0.929	4.16 (0.24–70.70)	0.324
Clerical service worker	0.74 (0.13–4–27)	0.737	3.88 (0.23–65.91)	0.349
Teachers	1.06 (0.18–6.15)	0.951	4.52 (0.26–77.21)	0.297
Retired	0.96 (0.17–5.48)	0.962	6.09 (0.37–101.5)	0.369
**Chronic systemic disease**				
Yes	1.18 (0.87–1.59)	0.293	1.27 (0.88–1.85)	0.204
No	1		1	
**Chronic eye disease**				
Yes	2.64 (1.42–4.88)	0.002	2.63 (1.34–5.19)	0.005*
No	1		1	
**Time using digital devices before lockdown in hours/day**				
1–2	1		1	
3–5	0.71 (0.51–1.0)	0.054	1.29 (0.74–2.25)	0.364
6–8	0.78 (0.58–1.0)	0.092	1.71 (1.07–2.72)	0.023*
>8	0.90 (0.64–1.26)	0.545	1.37 (0.80–2.34)	0.253
**Taking regular breaks**				
No	1		1	
Sometimes	0.74 (0.38–1.45)	0.385	0.98 (0.47–2.05)	0.955
Always	0.49 (0.23–1.02)	0.058	0.79 (0.35–1.81)	0.582
**Time increase using digital devices**				
Yes	3.56 (1.87–5.24)	0.019	5.16 (3.73–7.92)	0.000*
No	1		1	
**Time using digital devices during lockdown (hours/day)**				
= 0				
<1	1		1	
1–2	0.20 (0.14–0.29)	0.001*	3.43 (2.18–5.41)	0.001*
3–4	0.35 (0.25–0.48)	0.001*	6.23 (4.03–9.83)	0.001*
> 4	0.46 (0.34–0.62)	0.001*	9.06 (5.92–13.87)	0.001*

*Indicates statistically significant values.

This research showed that individuals with chronic eye problems are at a higher risk of developing new DES complaints and increasing the severity of their symptoms, even if they spend less time using digital. The results of Ranasinghe et al. (21) indicated that chronic eye diseases were the greatest risk factor for DES development among Sri Lankan computer workers (21). As well, both new and severe eye symptoms were associated with the presence of chronic systemic diseases such as hypertension, diabetes mellitus, dyslipidaemia, neurological disorders, and allergy, as shown in Table 3. The ocular symptoms of DES syndrome are classified into two groups, with the first being comprised of symptoms that are related to accommodation and that include blurred vision of near objects, blurry vision at a distance after using the computer, focusing difficulties between different distances, double vision, headaches, and neck and shoulder pain. The second is associated with dry eye and that included burning sensation, irritation, discomfort, sensitivity to bright light, eyestrain, and headaches (1). Eyestrain and headaches are linked to binocular visual stress and accommodation, in addition to their connection to dryness as in Table 2 (16, 27).

The most common symptom was neck and/or upper shoulder pain, followed by symptoms of accommodative dysfunction and to a lesser extent dry eyes. Prior to the lockdown, approximately 201 participants reported neck and/or shoulder pain; however, during the lockdown, this number nearly doubled to 390. This is in line with similar studies that found neck pain to be a common symptom among computer users, ranging from 19% to 70% (11–13, 28, 29). Touch screen devices, according to Kargar et al., necessitate more hand and head movements, resulting in arm/neck pain (30). Another study found that 68% of participants experienced musculoskeletal pain as a result of using touch screens, with neck pain and upper shoulder pain reported at 84.6% and 65.4 %, respectively, due to unnatural sitting positions without adequate back support (31). Logaraj et al., who found that 60.7 % of medical students reported neck pain as the most common symptom of DES, reported similar findings (25). Workers who used computers for more than 6 h per day were more likely to report upper limb disorders, according to Blatter and Bongers (19). As an unexpected mechanism to explain musculoskeletal pain is the oculomotor accommodative, and vergences dysfunction due to DES; electromyography has shown that ciliary muscle contraction is associated with head and neck muscle activation. The stabilization of gaze by head and neck muscles during accommodation was studied by Richter et al. (32). They noticed increased trapezius muscle activity in a dose-dependent manner when subjects were given different lenses in front of their eyes to stimulate the ciliary body. More head and neck muscle activity was observed, as accommodation was activated (32). Research studies provide similar findings, but the symptoms occur in a different order that is reflective of sampling and geographical variations (17, 18, 33, 34).

As expected, blurred vision, both near and far, was the second most common symptom reported at baseline and during the lockdown (6, 25, 35, 36). Rosenfield et al. attributed it to an incorrect accommodative response, as well as a failure to relax the ciliary body after the visual demand was completed (3). The use of smart phones and handheld devices, according to Jaiswal et al. (36), cause symptoms that are similar to DES because they stimulate the accommodative facility, resulting in decreased amplitude when the eye is fatigued. Despite the fact that no definitive evidence has been found linking smartphones to accommodative facility dysfunction, additional research is required to uncover the actual impact of digital devices on long-term users (36).

Dry eye symptoms might not be a legitimate component of DES, as dry eye disease may aggravate accommodative symptoms, especially in elderly men and women, as well as those who have ocular surface disease. However, DES affects people under the age of 18 who use digital devices; this necessitates the need to develop a more specific and precise definition of DES (37). Many patients who use dry eye treatments and increase their rate of blinking did not notice an improvement in their digital eye strain symptoms. Rosenfield and Jaiswal examined various factors that affect dry eye disease and its relation to DES in their reviews (3, 36). They identified that various environmental factors, such as humidity, ambient lighting, fans, blinking rate, corneal exposure to air, gender, age, medications, systemic diseases, contact lenses, tear film volume, osmolality, and tear film composition, all affect the development of dry eye disease. Nonetheless, DES is still affecting normal people who are not at risk for dry eye disease (16, 22, 38, 39).

### Limitations of the Study

The following limitations have been identified: To begin, the survey is based on a participant's self-report that has not been independently validated by clinical diagnostic testing. We avoided under- or over-estimating eye problems in our study by using an appropriate sample size and multivariate statistics. Second, the study did not include self-medication, medical, or environmental variables that may increase dry eye disease during lockdown. Third, an online survey has numerous limitations, including biased selection and the difficulty of measuring population changes over a single time period. However, the findings of this study established the lockdown effect on digital eye strain, particularly in Jordan.

## Conclusions

The results of this online survey reveal the negative effect of COVID-19 on home confinement with eye problems due to the significant increase in usage time of digital devices, which is also indicative of a more sedentary lifestyle. The results concur with recent studies demonstrating that lockdown could dramatically increase digital eye strain that became a growing public health issue that affects people of all ages and occupational groups, posing a threat to their health and quality of life.

Indeed, individuals who spent more time on digital devices developed a new eye complaint four times more often than who did not. Females are at higher risk of having severe symptoms. As the first in Jordan, this study could explore the impact of lockdown on developing DES-related eye symptoms. The visual consequences of the COVID-19 outbreak, which placed a curfew on people all over the world, should attract more attention from researchers these days. Neglecting DES could cause the exacerbation of mild symptoms in people who had them before or the emergence of new complaints in people who never had them previously. The government of Jordan would develop public health interventions to mitigate the negative effects of internet use on eye problems that have manifested during the COVID-19 lockdown.

## Data Availability Statement

The raw data supporting the conclusions of this article will be made available by the authors, without undue reservation.

## Ethics Statement

The studies involving human participants were reviewed and approved by IRB Committee at Al-Balqa Applied University. The patients/participants provided their written informed consent to participate in this study.

## Author Contributions

MA, HJ, and HA: conceptualization, data curation, data analysis, writing—original draft preparation, and writing—reviewing and editing. MA and HJ: methodology. MA: supervision. All authors have agreed to the order of authorship and approved the submission of this version and are accountable for the content of this manuscript.

## Conflict of Interest

The authors declare that the research was conducted in the absence of any commercial or financial relationships that could be construed as a potential conflict of interest.

## Publisher's Note

All claims expressed in this article are solely those of the authors and do not necessarily represent those of their affiliated organizations, or those of the publisher, the editors and the reviewers. Any product that may be evaluated in this article, or claim that may be made by its manufacturer, is not guaranteed or endorsed by the publisher.

## Abbreviations

DES: digital eye strain
VDT: video display terminal.
